# Bioenergetic failure correlates with autophagy and apoptosis in rat liver following silver nanoparticle intraperitoneal administration

**DOI:** 10.1186/1743-8977-10-40

**Published:** 2013-08-19

**Authors:** Tzu-Ying Lee, Maw-Shung Liu, Li-Ju Huang, Sheng-I Lue, Lung-Chang Lin, Aij-Lie Kwan, Rei-Cheng Yang

**Affiliations:** 1 Graduate Institute of Medicine, College of Medicine, Kaohsiung Medical University, Kaohsiung 807, Taiwan; 2 School of Medicine and Health Sciences, Foo-Yin University, Kaohsiung 807, Taiwan; 3 Department of Physiology, College of Medicine, Kaohsiung Medical University, Kaohsiung 807, Taiwan; 4 Department of Pediatrics, Kaohsiung Medical University Chung-Ho Memorial Hospital, Kaohsiung 807, Taiwan; 5 Department of Neurosurgery, Kaohsiung Medical University Chung-Ho Memorial Hospital, Kaohsiung 807, Taiwan; 6 Center of Excellence for Environmental Medicine, Kaohsiung Medical University, Kaohsiung 807, Taiwan; 7 Department of Pediatrics, Changhua Christian Hospital, Changhua county 526, Taiwan

**Keywords:** Silver nanoparticles, ATP, Autophagy, Apoptosis

## Abstract

**Background:**

Deposition and accumulation of silver nanoparticles (Ag-nps) in the liver have been shown to induce hepatotoxicity in animal studies. The hepatotoxicity may include oxidative stress, abnormalities in energy metabolism, and cell death. Studies have indicated that autophagy is an intracellular event involving balance of energy, nutrients, and turnover of subcellular organelles. The present study was undertaken to test the hypothesis that autophagy plays a role in mediating hepatotoxicity in animal after exposure to Ag-nps. Focus was placed on interrelationship between energy metabolism, autophagy, apoptosis and hepatic dysfunction.

**Methods:**

Sprague Dawley rats were intraperitoneally injected with Ag-nps (10–30 nm in diameter) at concentration of 500 mg kg^-1.^ All animals were sacrificed on days 1, 4, 7, 10 and 30 after exposure and blood and liver tissues were collected for further studies.

**Results:**

Uptake of Ag-nps was quite prompt and not proportional to the blood Ag concentration. Declination of ATP (-64% in days 1) and autophagy (determined by LC3-II protein expression and morphological evaluation) increased and peaked on the first day. The ATP content remained at low level even though the autophagy has been activated. Apoptosis (based on caspase-3 protein expression and TUNEL-positive cells staining) began to rise sigmoidally at days 1 and 4, reached a peak level at day 7, and remained at the same levels during days 7–30 post exposure. Meanwhile, autophagy exhibited a gradual decrease from days 1–10 and the decrease at day 30 was statistically significant as compared to day 0 (sham group). Inflammatory reaction (histopathological evaluation) was found at day 10 and preceded to an advanced degree at day 30 when liver function was impaired.

**Conclusions:**

These results indicate that following Ag-nps administration, autophagy was induced; however, failure to preserve autophagy compounded with energy reduction led to apoptosis and the eventual impairment of liver function. The study provides an *in-vivo* evidence of hepatotoxicity by continuous exposure of Ag-nps in rats.

## Background

Nanoparticles are defined as particles having a diameter smaller than 100 nm [1]. Due to their unique physico-chemical properties, nanoparticles are widely used in numerous aspects such as chemical industry, biotechnology, environmental technology and biomedicine [2]. Among products available in the market, those containing silver nanoparticles (Ag-nps) are the largest and the fastest growing category because of their unique characteristics of antibacterial activities [3-5]. Currently, manufactured products containing Ag-nps may include wound dressings, drugs, clothing, cosmetics, bedding, water purification, washing machines, deodorants, and humidifiers. With increasing applications of Ag-nps containing products, it is important to study their adverse effects.

Ag-nps have been reported to enter the body through inhalation, ingestion, injection and dermal contact, resulting in a dose-dependent increase of silver concentration in various organs in animal studies [6-8]. Most foreign Ag-nps were found to accumulate in the liver, a major organ of detoxification [8,9]. Excessive accumulation/deposition of Ag-nps in the liver caused certain adverse effects including marked pathological changes in liver morphology, bile-duct hyperplasia, inflammatory cell infiltration [10-12], generation of excessive reactive oxygen species, DNA damage, changes in liver enzyme activities [9] and finally leading to apoptosis and necrosis [11,13]. However, while facing an adverse situation, cells execute a homeostasis mechanism, *i.e.*, autophagy, to promote cell survival [14-16]. Autophagy is a conservative intracellular protein degradation system that consists of several sequential steps. In brief, after formation of double-membrane-enclosed autophagosomes, the autophagosome engulf cytoplasmic misfolded proteins, injured and unwanted organelles, and subsequently deliver them to lysosomes for digestion. Several key molecules are involved in autophagy process, especially the processing of microtubule associated protein 1-light chain 3-II (LC3-II) which is regarded as an autophagosome biomarker [17]. This phenomenon has been found to take place under both normal and pathophysiological situations, including cell survival, cell death, cell metabolic stress, development, infection and immunity, and aging [18]. On the other hand, it has also been reported that the prolonged autophagy increased cellular stress and directly or indirectly induced cell death through excessive self-digestion and activation of apoptosis [19]. Therefore, the aim of the present study was to investigate sequential changes of autophagy and apoptosis and their relationship to energy homeostatic state in rat liver after an intraperitoneally injection of Ag-nps.

## Results

### Transmission electron microscopic (TEM) observation of Ag-nps

**Figure 1:**
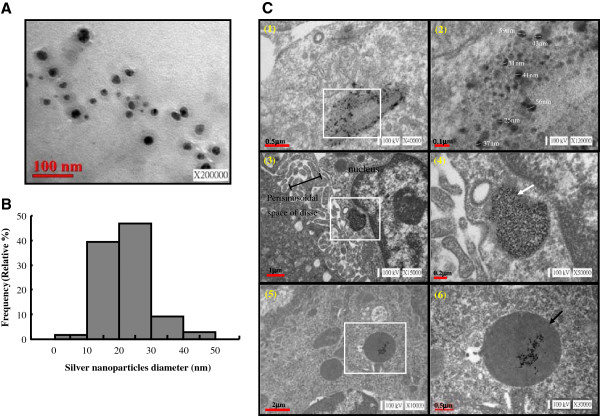
**Characterization of Ag-nps.** Size distribution of Ag-nps following dispersion in water **(A-B)** and their distribution in suborganelles of liver tissues obtained from rats at day 30 following Ag-nps administration **(C)**. Tissues were processed for TEM evaluation as described under Materials and Methods. Accelerating voltages and magnifications were indicated at right lower corner. **A-B:** particle size distribution evaluated from the corresponding TEM micrograph (n = 109). **C:** scale bars size represent 0.5 μm in panels 1 and 6, 0.1 μm in panel 2, 1 μm in panel 3, 0.2 μm in panel 4, and 2 μm in panel 5. Panel 2 represents the enlargement of white square area indicated in panel 1. Panel 4 and 6 represents the enlargement of white square area indicated in panel 3 and 5, respectively. White dotted lines in panel 2 indicate diameters of Ag-nps. White arrow in panel 4 indicates endosome. Black arrow in panel 6 indicates lysosome.

Figure 1 show particle deposition of Ag-nps in liver tissues (Figure 1C) and size distribution of Ag-nps following dispersion in water (Figures 1A-1B). As shown in Figure 1A and 1B, the TEM images reveal that all nanoparticles were not appeared in aggregated form and they were rounded in shape (Figure 1A). Approximately 86% of the dispersed particles were in sizes ranging from 10–30 nm in diameter (Figure 1B). The mean diameter of the dispersed Ag-nps was 22.32 ± 7.07 nm ( M ± SD, n = 109). These data demonstrate that under our experimental conditions, the suspended Ag-nps were in the individual form with a uniform size distribution. As shown in Figure 1C (Panels 1–6), most of the intracellular Ag-nps appear to be rounded in shape with a diameter ranging from 25–59 nm in hepatocyte. Meanwhile, Ag-nps were not appear in hepatocyte of sham group (Additional file 1). The distribution of the Ag-nps was confined to and localized in endosome (Panel 4) and lysosome (Panel 6) of the cytoplasm. These results demonstrate that Ag-nps deposited in the liver and distributed in endosomal-lysosomal compartments. It should be noted that the deposition of Ag-nps in other cell type, *i*.*e*. Kupffer cells was also observed (Additional file 2).

### Silver concentration in liver tissue and whole blood

**Figure 2:**
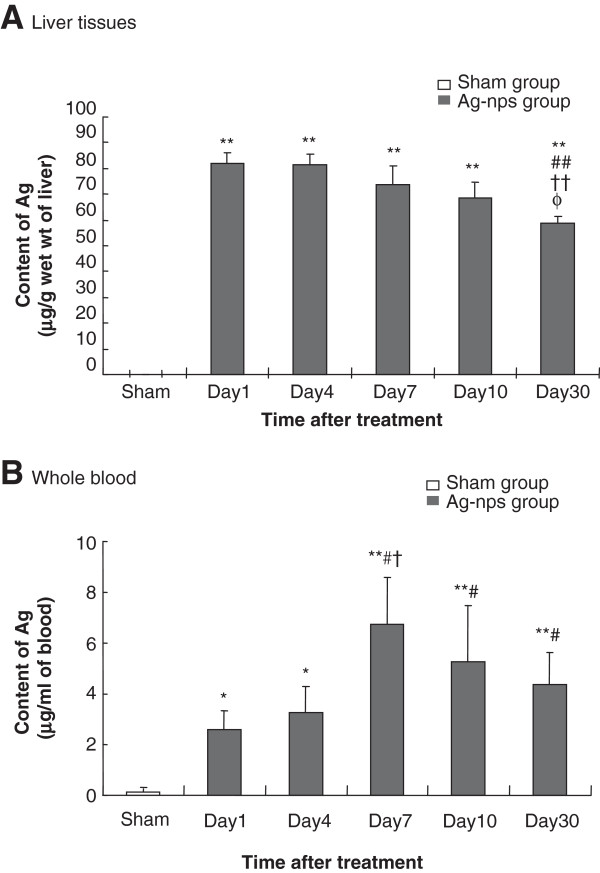
**Time-course of silver deposition in liver tissues (A) and whole blood (B) following Ag-nps administration.** Ag-nps were intraperitoneally injected at a dose of 500 mg kg^-1^ for 1, 4, 7, 10 and 30 days. Liver tissues and whole blood samples were processed for ICP-MS analysis as described under Materials and Methods. Empty columns represent sham groups while filled columns represent Ag-nps treatment groups. Vertical bars indicate standard deviations of the mean. Number of experiments was 8 for each time points. ^*^p < 0.05, ^**^p < 0.01 vs. Sham; ^#^*p* < 0.05, ^##^*p* < 0.01 vs. Day 1; ^†^*p* < 0.05, ^††^*p* < 0.01 vs. Day 4; ^φ^*p* < 0.05 vs. Day 7.

Figure 2 depicts the time course of silver deposition in liver tissues and whole blood following *i.p.* administration of Ag-nps. In sham groups, small but measurable amounts of silver were detected in the liver and whole blood (0.10 ± 0.23 μg/g for liver; 0.05 ± 0.06 μg/ml for blood) (Figure 2). In treatment groups, deposition of silver in the liver were found to be highest at days 1 and 4 (in wet weight of liver; 81.84 ± 4.37 for day 1; 81.36 ± 4.26 for day 4) and reduced gradually from day 7 to day 30. However, it retained at a high level at day 30 (58.76 ± 2.84 μg/g wet weight). These findings indicate that Ag-nps were rapidly absorbed into liver cells. The slow decrease in silver concentrations (~28.20%) from day 1 to 30 may indicate a time-dependent increase in excretion. When the same data were expressed as fractional deposition of the administered dose, similar results were obtained. In whole blood (Figure 2B), silver concentrations were increased from day 1 (2.62 ± 0.70 μg/ml) and peaked concentration at day 7 (6.76 ± 1.83 μg/ml) and then declined moderately until day 30. The results from Panel A and B indicate that dissociation in time consumption reaching maximal level was present between liver tissue and circulating blood.

### Liver function changes

Table 1 shows changes in the enzymatic activities of AST and ALT in serum at various time points following *i.p.* administration of Ag-nps. AST activities were significantly augmented in serum at each time point (day 1, 4, 7, 10, and 30) during the entire course of experiment following an *i.p.* injection of Ag-nps. ALT activities were decreased at days 1 and 4 while they were increased at day 30. These results indicate that at the concentration (500 mg kg^-1^ body weight) of Ag-nps administered, liver function was affected during the course of study.

**Table 1:** Time course of changes in serum enzyme activities in rats following intraperitoneal administration of Ag-nps

**Time after treatment (day)**	**Group**	** AST (U/L)**	**ALT (U/L)**
1	Sham	84.00 ± 27.46 **	45.93 ± 12.70**
	Ag-nps	128.13 ± 40.99	25.68 ± 10.72
4	Sham	84.00 ± 27.55 *	49.13 ± 11.80**
	Ag-nps	99.24 ± 20.90	36.09 ± 13.21
7	Sham	87.17 ± 16.82 **	51.31 ± 11.63
	Ag-nps	131.13 ± 37.40	50.71 ± 10.03
10	Sham	86.75 ± 10.01**	50.00 ± 11.07
	Ag-nps	157.15 ± 47.03	50.15 ± 13.28
30	Sham	94.67 ± 13.58*	48.50 ± 6.81*
	Ag-nps	139.25 ± 11.69	58.44 ± 12.18

Values were mean ± standard deviations of the mean. N = 8 at each time point for each group. Ag-nps were administered, *i*.*p*., at 500 mg kg^-1^ body weight. AST = aspartate aminotransferase; ALT = alanine aminotrasferase. *p < 0.05, **p < 0.01 as compared to sham groups.

### Intrahepatic distribution of Ag-nps

**Figure 3:**
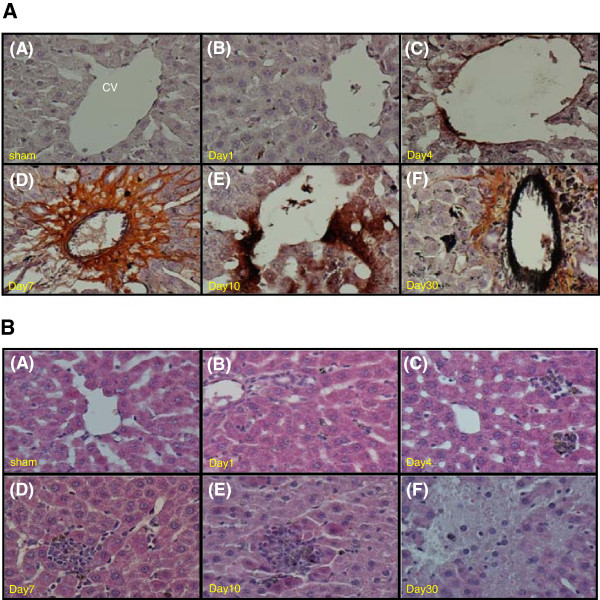
**Histopathological observation of rat liver.** Intrahepatic distribution of Ag-nps **(A)**, and histopathological changes in liver tissues at different time points **(B)** after *i.p.* injection of Ag-nps. Experiments were carried out as described under Materials and Methods using silver enhancement stain and hematoxylin and eosin stain. **A:** section thickness was 5-μm. Ag-nps images were taken by optical microscopy with 200× amplifications. **B:** high magnification of livers (400×).

Figure 3A shows intrahepatic distribution of Ag-nps over a period of 30 days following *i.p.* injection of Ag-nps in rats. Based on the silver enhancement procedure, the formation of brown/black signals are indicative of the precipitation of metallic silver. Silver-stained signals were increased consecutively from day 1 to day 30 (Panel B to F), and the signals were mostly located around the blood vessels. These results indicate that the accumulation/deposition of Ag-nps was time-dependent and most prominently occurred during days 4 to 7. Furthermore, the results indicate that tissue accumulation/deposition is a result of diffusion of Ag-nps from portal vein into liver cells. It should be noted that the time-dependent accumulation/deposition of Ag-nps as shown in this figure paralleled with intravascular (whole blood) concentrations of silver as depicted in Figure 2B.

### Histopathological examination of liver tissues

Histopathological evaluations of liver tissues were conducted under light microscopy with H&E stain as depicted in Figure 3B. At day 1, no change of histological characteristics of liver tissues was observed in Ag-nps group as compared to the sham group. At days 4–7, there was no major changes in the structural component of the liver while occasional foci of inflammatory cell infiltrates were present (Panel C and D). At day 10, foci of liver cell degeneration appeared in addition to moderate inflammatory cell infiltration (Panel E). At day 30, liver cell degeneration became prominent and it was accompanied by evidence of piecemeal necrosis and chronic inflammatory cell infiltration. These results indicate that following an *i.p.* injection of 500 mg kg^-1^ of Ag-nps, an inflammatory reaction was induced in the liver beginning at day 4, and the inflammation was proceeded modestly during days 7–10, and then to a more advanced stage at day 30.

### Induction of autophagy after Ag-nps administration

**Figure 4:**
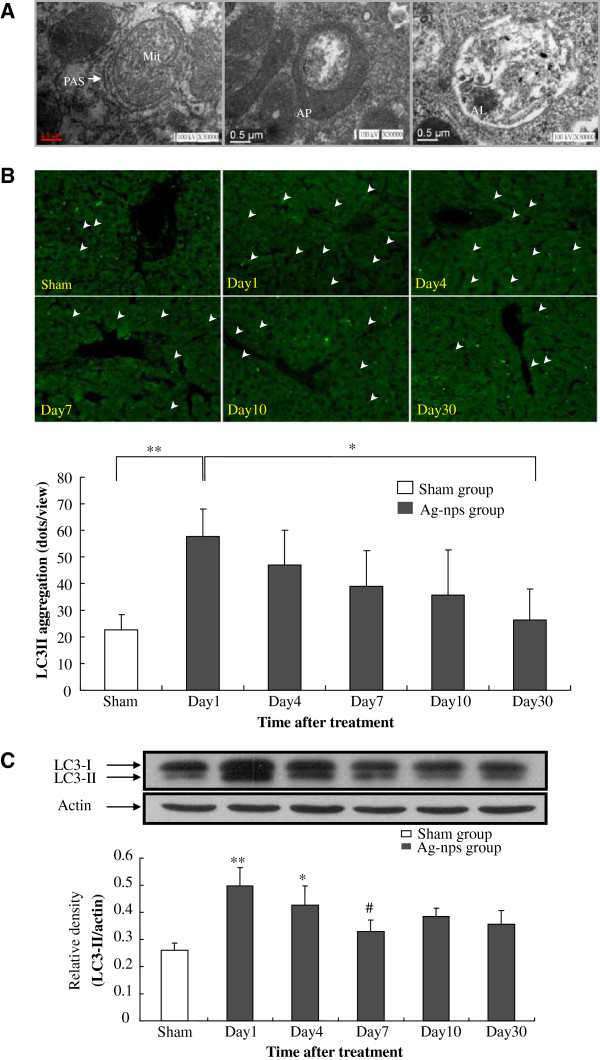
**A transient increase of autophagy in rat liver.** Changes in autophagic structures (day 1) **(A)**, LC3-II aggregation (day 1–30) **(B)**, and LC3-II protein expression (day 1–30) **(C)** in liver tissues at various time points following an *i.p.* Injection of Ag-nps. Experiments were carried out as described under Materials and Methods. **A:** TEM images of rat liver tissues. Scale bar = 0.5 μm. PAS = phagophore assembly site; Mit = mitochondria; AP = autophagosome; AL = autophagolysosome. **B:** upper panel: LC3-II aggregation was analyzed by immunofluorescence staining under fluorescence microscopy. Lower panel: quantitative analysis of FITC-LC3-II aggregation from each time points was estimated by counting the number of bright green dots, as arrows indicated (upper panel), under 200x amplification. Data were means ± SD of 8 individual rat experiment, *p < 0.05, **p < 0.01. **C:** LC3-II protein level changes based on Western blot analysis. Actin was used as a loading control. Values were expressed as means ± SD (n = 6), *p < 0.05 and **p < 0.01 versus sham group; ^*#*^*p* < 0.05 versus Day 1 group.

Figure 4 depicts changes in autophagic structures (day 1) (Figure 4A), LC3-II aggregation (day 1–30) (Figure 4B), and LC3-II protein expression (day 1–30) (Figure 4C) in liver tissues at various time points following an *i.p.* injection of Ag-nps. The TEM examination (Figure 4A) reveals that phagophore structure, double-membrane autophagosome with engulfed damaged organelles, and autolysosome with a large vacuole containing large amount of cellular debris were all present in liver tissues. Autophagic vacuoles (AV) also present in macrophage, in addition to, hepatocytes (Additional file 3). These results demonstrate the autophagic structures in liver cells were induced at day 1 following Ag-nps administration. The induction of autophagy was confirmed by immunofluorescence evaluation of liver tissues (Figure 4B). As shown in Figure 4B, scant green FITC-LC3-II punctate dots were observed in sham group whereas significant increases in LC3-II punctate dots were observed at day 1 (*p* < 0.01) following Ag-nps treatment. The LC3-II punctate dots were reduced from day 4 and the reduction was continues till day 30. The induction of autophagy was further supported by changes in the expression of LC3-II (Figure 4C), a protein widely used as a hallmark of autophagy. As shown in Figure 4C, LC3-II protein expression was significantly increased at day 1 as compared to sham group (*p* < 0.01). The LC3-II protein expression was gradually declined thereafter in a time-dependent manner. The results from TEM studies (Figure 4A), LC3-II aggregation (Figure 4B), and LC3-II protein expression (Figure 4C) unequivocally demonstrate that the autophagy was induced at vary early stage following Ag-nps administration and it was gradually diminished thereafter.

### Evaluation of apoptosis

**Figure 5:**
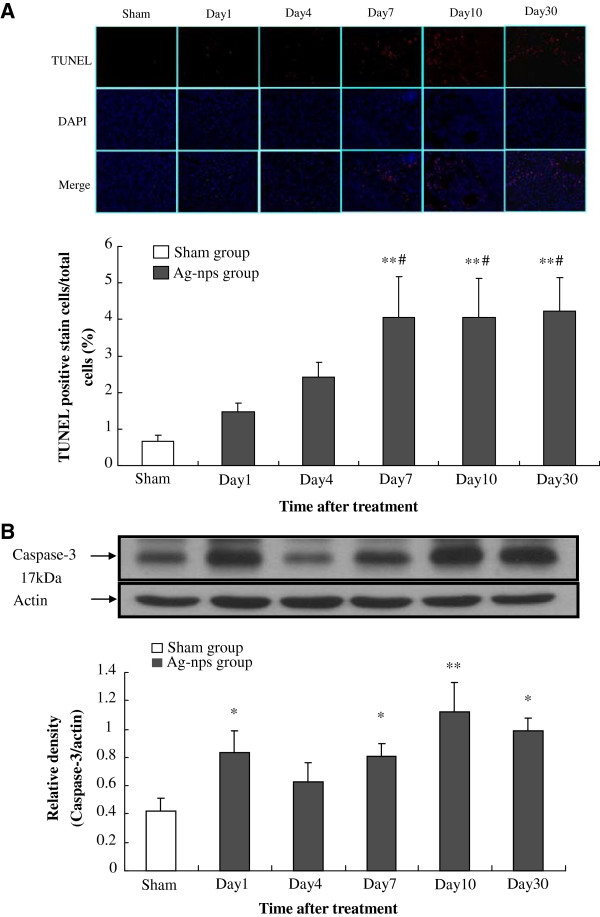
**Level of the apoptosis was observed in hepatocytes. A:** Fluorescence images of TUNEL staining (red) and DAPI staining (blue) in liver tissues at various time points following Ag-nps administration. Experiments were carried out as described under Materials and Methods. Upper panel shows representative histograms of TUNEL and DAPI staining while lower panel depicts quantitative analysis of TUNEL/DAPI positive cells. Values (means ± SD) presented in lower panel were obtained by dividing numbers of TUNEL positive cells with total numbers of cells from DAPI stain. Number of experiments was 6 for each time point. **p < 0.01 vs. sham group; ^#^*p* < 0.05 vs. Day 1 group. **B:** Changes in caspase-3 protein expression in rat liver at various time points following Ag-nps administration. The experiments were performed as described under Material and Methods. Upper panel shows the representative immunoblots of caspase-3 while lower panel depicts densitometric analysis of immunoblots. Values were presented as means ± SD (n = 6). *p < 0.05 and **p < 0.01 compared to sham group.

To further assess the extent of cell death by Ag-nps, apoptosis was evaluated by fluorescence analysis based on TUNEL and DAPI staining (Figure 5A), and Western blot examination of caspase-3 protein expression (Figure 5B). As shown in Figure 5A, significant increases of TUNEL-positive cells were detected in a time-dependent manner in Ag-nps administered group while few TUNEL-positive cells were found in sham group. Increases of apoptotic cells were observed at days 1 and 4 (1.47 ± 0.23% for day 1; 2.42 ± 0.41% for day 4), reached a peak level at day 7 (4.06 ± 1.11%), and remained at the same levels at days 7, 10, and 30 after Ag-nps treatment, as compared to sham group (0.67 ± 0.15%). These results strongly indicate that apoptosis took part in the mechanism of toxicity by Ag-nps. As shown in Figure 5B, Ag-nps caused a significant increase in caspase-3 protein levels in the liver of treated rats. Since caspase-3 is an intracellular cysteine-aspartic acid protease and a well-established cellular marker in the initiation and the execution of apoptosis, the results depicted in Figure 5B suggest that the intrinsic pathway was involved in the rat liver apoptotic cell death upon administration of Ag-nps.

### Hepatic ATP depletion after Ag-nps administration

**Figure 6:**
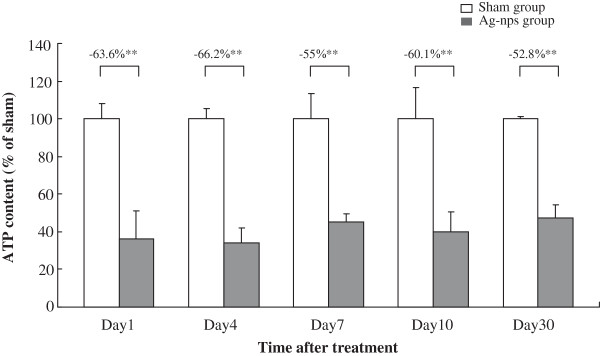
**Time course of changes in ATP content in rat liver tissues following Ag-nps administration.** ATP contents in liver tissues were determined by luciferin-luciferase assay as described under Material and Methods. Values were presented as means ± SD (n = 4-6). **p < 0.01 compared to the corresponding sham group.

Figure 6 depicts time course of changes in ATP content in liver tissues following Ag-nps administration. There was a significant reduction (-63.6%) in ATP content of livers at day 1 after Ag-nps treatment. The ATP content remained at the reduced levels (-52.8% to -66.2%) throughout the entire experimental period (days 4–30). These results indicate that ATP may play a critical role in the induction of hepatic toxicity following Ag-nps administration.

## Discussion

**Figure 7:**
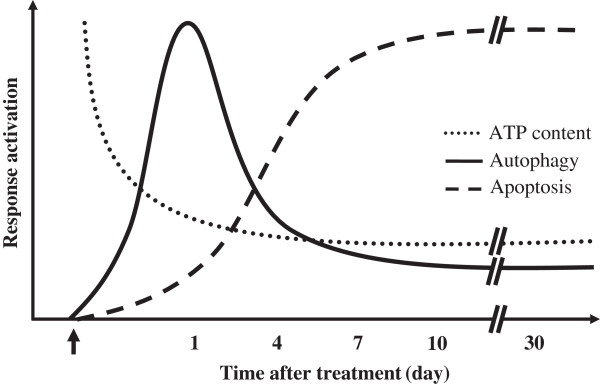
**Interrelationship among changes in ATP content, autophagy, and apoptosis in rat livers after Ag-nps administration.** This figure was constructed using data from Figures 4, 5, and 6.

In this study, we aimed to investigate interrelationship between changes in energy metabolism, autophagy, apoptosis and hepatic dysfunction in rats upon exposure to Ag-nps as illustrated in Figure 7. Review of literature shows that animals were exposed to Ag-nps by different methods including intravenous, oral, inhalation and intraperitoneal administration. Tiwari et al. [9] reported that following intravenous injection, the concentration of silver in the liver was 18 μg/g. Loeschner et al. [7] found that following oral exposure, the level of silver deposited in the liver was less than 1 μg/g. Sung et al. [12] reported that following inhalation, the amount of silver accumulated in the liver was 0.133 μg/g. In our study, we found that following intraperitoneal injection, the concentration of silver in the liver was 58–81 μg/g. Putting these data together, it is clear that intraperitoneal injection has achieved the highest tissue deposition of Ag-nps in the liver as compared to other routes of administration, *i.e*. intravenous, oral, and inhalation. Intraperitoneal administration has been widely used for studies of the accumulation and the resorptive toxicities of nanoparticles including nanosilver [20,21], nanogold [21], and nano-Fe_3_O_4_[22]. Furthermore, most foreign Ag-nps were found to accumulate in the liver, the major organ of detoxification [8,9]. Accordingly, the intraperitoneal administration was then adapted in this studies.

Organ accumulation has been demonstrated with various nanomaterials [23]. Among various organs, liver is the main target of Ag-nps in addition to all blood derived antigens [8,24]. Our histological observation reveals that Ag-nps mainly displayed around the blood vessels, indicating that Ag-nps was absorbed through mesenteric vein via portal system and then distributed into hepatic tissues. The reactivity to silver staining surrounding vessels became apparent from day 7 after exposure, suggesting that the endothelial barrier started to collapse and subsequent massive injury occurred to liver cells.

In our study, the deposited Ag-nps in hepatocytes were found to be individual particles with a size smaller than 100 nm in diameter. Examination of TEM images reveal that Ag-nps were localized predominantly in endosomes and lysosomes of hepatocytes. These findings provide evidence to corroborate previous *in-vitro* reports describing the uptake of Ag-nps occur mainly through endocytosis. In addition to hepatocyte, Ag-nps were found to be accumulated in macrophages (Kupffer cells). The Ag-nps in Kupffer cells were in agglomerates (> 100 nm), indicating partial agglomeration of Ag-nps after cell internalization. These observations demonstrate that endosome and lysosome compartments are the ultimate fate of Ag-nps for deposition and degradation, in the liver. We have also observed in our study that Ag-nps deposited in Kupffer cells while hepatocytes exhibited mild infiltration of inflammatory cells in portal vein area (Figure 3B). These findings indicate that Kupffer cells were involved in the process of inflammation following Ag-nps exposure. Further studies may shed light on the precise role of Kupffer cells in the Ag-nps mediated hepatic injury.

Following *i.p.* injection, a rapid and maximal increase of silver concentration was detected as soon as day 1 in the liver, indicating that Ag-nps were precipitously absorbed into liver tissues. The hepatic silver concentration showed a slight decline thereafter in which 72% of silver were still retained towards day 30. However, the accumulation of silver in the blood peaked at a later time, *i.e*., day 7. The difference in the time course of peak concentrations of silver in the liver and blood suggests that blood concentration may not be a reliable indicator of organ storage for Ag-nps and should be cautious in clinical practice.

Most of the administered nanoparticles have been reported to be excreted from kidney or hepatobiliary pathways within 15 days [25,26]. In case of Ag-nps, we found that Ag-nps failed to be cleared completely from the body within 30 days. There are several possibilities which could account for this event. The formation of silver-protein complexes due to strong binding of Ag-nps to thiol groups of various protein moieties that diminish Ag expulsion. Alternatively, combination of silver with DNA bases in the nucleus [27,28], or the interference on the activation of autophagy/exocytotic processes due to failure of bioenergetic supply could slow the elimination process [29].

Hepatic function was evaluated by measuring AST and ALT [30]. The fact reveals that serum ALT and AST levels were both elevated till day 30, indicating that liver tissues were damaged at late stage following Ag-nps administration. The less than overwhelming activation, *i.e*., increase in AST activation during days 1 to 10 accompanied with decrease in ALT activation during days 1 to 4, suggests that liver tissues were less damaged during the early and mid stages of experiments. This notion is supported by histological findings that no significant alterations were observed during early stage, and that evidence of damages including apoptosis and necrosis appeared in a later stage.

Lack of biochemical and morphological changes observed in our study during early stage of Ag-nps exposure does not necessarily indicate a muted cellular response. Perceived as a foreign material, nanoparticles lead cells to initiate a self-protective mechanism by induction of autophagy. The *in-vitro* studies show that gold and TiO_2_ nanoparticles induced autophagy as a defensive mechanism in human fibroblasts and cerebral endothelial cells within 2–3 days [31,32]. However, no *in-vivo* study, to be best of our knowledge, has been reported. In this study, autophagy was induced in liver tissues 1 day after Ag-nps exposure at which time tissue concentration of silver was at its maximal level. The proficiency of autophagy decelerated gradually within days and weeks while tissue accumulation of Ag-nps remained high. Interestingly, apoptosis of hepatocytes began to rise sigmoidally along with the declination of autophagy by evidence of increased expression of its marker protein, caspase-3 and TUNEL-positive cells identification. It implies that the decline in autophagy along with a high concentration of silver may cause insufficient self-protection, which contributes to cell damage. It is noteworthy that increasing number of reports has demonstrated that autophagy disturbances, either over-induction or inhibition, can be responsible for the autophagy dysfunction-mediated apoptosis [33]. In this study, we found that LC3-II protein expression was diminished during mid and late stages, indicating that the observed autophagy in the liver was not a result of over-induction.

Mitochondrial dysfunction has been established to be a sensitive target of oxidative stress-induced cytotoxicity and genotoxicity by Ag-nps [27]. Ag ions have been reported to cause disturbance and destruction of mitochondrial function through interaction with thiol groups of inner mitochondrial membrane proteins [34]. Moreover, it has been reported that Ag-nps decrease the activity of mitochondrial respiratory chain complexes and reduce antioxidant factors like glutathione, thioredoxin, superoxide dismutase and *N*-acetylcysteine in liver cells [11,35]. In response to these changes, cells induce compensatory pathways including autophagy. Autophagy is the primary mechanism for removal of damaged organelles, such as mitochondria. Rikiishi [36] reported that in periods of metabolic stress, autophagy provides ATP and other macromolecules as energy sources to enable cell survival. In this study, we found the ATP content decreased on the first day after exposure to Ag-nps and the decreased persisted till the end of the experimental period. It is apparent that energy synthesis was perturbed during early phase following Ag-nps administration and the persistence on the impaired energy metabolism eventually leading to deceleration of autophagy and acceleration of apoptosis.

Ag-nps dispersed in aqueous medium release Ag ions. It is necessary to distinguish toxic effects between Ag-nps and the dissolved Ag ions. Ag-nps and the released ions readily bind to proteins and DNA, thereby potentially causing cell damage. Pratsinis et al. [37] separated the released Ag ions from the particles and measured their toxicity; they found that the observed toxicity was a function of nanoparticle size. The size of the Ag-nps dictates its mode of cytotoxicity, *i.e.*, Ag ion-specific and/or particle-specific. Smaller Ag-nps release or leach larger fractions of their mass as Ag ions upon dispersion in water. The toxicity of small Ag-nps (<10 nm) is mostly mediated by the released Ag ions due to its larger surface area per unit mass and its higher Ag ion concentrations. For large Ag-nps (>10 nm), from which fewer Ag ions are released, the toxicity is attributed primarily to the dispersed particles rather than their initially released ions. Furthermore, Kim et al. [38] demonstrated that the cytotoxicity of Ag-nps is primarily the result of oxidative stress and is independent of the toxicity of Ag ions in human hepatoma cells. Nevertheless, we cannot rule out possible involvement of Ag ions in the Ag-nps-mediated toxicity observed in our studies.

It is noteworthy that the accumulation and the toxicity of nanomaterials in humans happen insidiously. Experimental assessment of nanotoxicology performed *in-vivo* by using appropriate dosing amounts and routes of exposure may carry greater significance because of the diversity of systemic phenotypic response and the physiologic/anatomic influence that can be translated from animal models to human exposures. In this study, in order to facilitate observation of experimental purposes, we selected the route of exposure and the dose that could be tolerated in the rat for experimental duration. The high dose used in our study may not necessarily reflect actual tissue levels of Ag-nps found in human organs, nevertheless, our results provide timely information fulfilling the gap in regard to the deficiency in the rapidly evolving area of human exposure to silver nanoparticles.

## Conclusions

In conclusion, we provide an *in-vivo* evidence of hepatic toxicity caused by continuous absorption of Ag-nps in rats. The 30-day observation period revealed that following Ag-nps administration, ATP content (cellular energy state) was decreased rapidly while autophagy was induced as a defensive mechanism. However, failure to preserve autophagy was compounded with bioenergetic defect which eventually lead to apoptosis and impaired liver function.

## Methods

### Materials

Silver nanoparticles (The physical characteristics of the particles according to the manufacturers data are; size (≤100 nm), purity (99.5% trace metal basis), surface area (5.0 m^2^/g), density 10.49 g/cm^3^ (lit.), Polyvinylpyrrolidone (PVP) as dispersant [19,39], anti-LC3B antibody, epoxy resin and DAPI (4′, 6′-diamidino- 2-phenylindole) were purchased from Sigma-Aldrich (St. Louis, MO, USA). A cleaved caspase-3 primary antibody was obtained from Cell Signaling Technology (Danvers, MA, USA). Fluorescein isothiocyanate (FITC)-conjugated AffiniPure goat anti-rabbit IgG were supplies by Jackson ImmunoResearch Laboratories (West Grove, PA, USA). Anti-β-actin mouse monoclonal antibody was purchased from Santa Cruz Biotechnology, Inc. (Delaware Avenue, California, USA). ECL Rabbit IgG, HRP-linked whole antibody and goat polyclonal secondary antibody to mouse IgG were obtained from GE Healthcare Life Sciences and Abcam (Pittsburgh, PA, USA and Cambridge, MA, USA), respectively. Anti-fade fluorescent mounting medium was supplies by DakoCytomation (Carpinteria, CA, USA). HQ (high quality) silver enhancement kit was obtained from Nanoprobes (Yaphank, NY, USA). ATP determination kit was supplied by Molecular Probes (Paisley, UK). In situ cell death detection kit, TMR red was purchased from Roche Applied Science (Mannheim, Germany). Other chemicals and regents were of analytical grade.

### Animal model and administration of Ag-nps

Male Sprague–Dawley rats (300–350 g; BioLasco Taiwan Co., Ltd) were used for our study. All animal experiments in this study were conducted with the approval of the Animal Care Committee of the Kaohsiung Medical University. Rats were randomly divided into two groups: sham-operated and treatment groups. Number of experiment was 6–8 for each group. Ag-nps were dispersed in deionized water to a concentration of 150 mg / 1.5 ml by vigorous vortexing followed by sonication for 5 min (amplitude of vibration were 10 microns and the frequency of vibration 46 kHz) to ensure uniformity. Under light ether anesthesia the treatment group received an intraperitoneally (*i.p.*) injection of Ag-nps preparation at a dose of 500 mg kg^-1^[22]. Only one dose was given during the entire experimental period. Preliminary experimented were performed to determine the dosage. A single *i.p.* injection of 1000 mg kg^-1^ of Ag-nps resulted in a 20% mortality at day 30 in rats (data not included). When the dose was reduced from 1000 mg kg^-1^ to 500 mg kg^-1^, the mortality was reduced to zero (0). Furthermore at day 30, there was no elevation of serum bilirubin level (Additional file 4) indicating that liver function remained intact. During the time course of study (days 1–30), the serum ALT level remained constant throughout the experimental period until the day 30. These findings indicate that the dosage (500 mg kg^-1^) adapted was appropriate for allowing us to study hepatotoxicity without harming hepatic function. Sham-operated groups received equal volume of deionized water, and time-matched. The values obtained at different time points (1, 4, 7, 10 and 30 days) for sham groups were virtually identical. The animals were then sacrificed at 1, 4, 7, 10 and 30 days following treatment. Blood samples and liver tissues were collected at each time point for biochemical and histopathologic analyses.

### Measurement of silver concentration in whole blood and liver tissues

Silver concentrations in liver tissues and whole blood were determined based on quantification of ^107^Ag using an inductively coupled plasma-mass spectrometry (ICP-MS) with modification [8]. In brief, prior to the elemental analysis, samples of whole blood (500 μl) and liver tissues (300 mg) were digested in 5 ml nitric acid for 3 days followed by the addition of 0.5 ml hydrogen chloride for 2 days. The acidic digested solution was diluted to a total volume of 25 ml with deionized water. Concentrations of silver in the samples were analyzed by ICP-MS (Thermo XSeries-II, Germany). The detection limit of silver was 0.007 μg L^-1^. To ensure the accuracy and precision of the technique, the indium was used as an internal standard.

### TEM studies

Ag-nps were characterized with TEM. After sonication for 5 min, samples were prepared by spraying homogeneous suspensions of Ag-nps on a carbon film-coated Cu grids and allowing it to dry in air. All images were taken at 200000× magnification and an accelerating voltage of 100 kV. Liver tissues were dissected into 1-mm^3^ pieces, and then immersed in a fresh 2% paraformaldehyde mixed with 2.5% glutaraldehyde in 0.1 M PBS (phosphate- buffered saline) overnight. The samples were post-fixed in 2% osmium tetroxide for 2 h at 4°C and dehydrated with ascending grades of alcohol. The tissue block was then infiltrated and embedded in epon resin at 60°C for 72 h. Ultrathin sections (70 nm) were cut with an automatic ultra-microtome (Reichert Ultracut E, Vienna, Austria) using a diamond knife. The sections were collected on copper grids (200 meshes) and stained with uranyl acetate and lead citrate solutions. TEM images were observed under a transmission electron microscope (JEM2000 EXII; Jeol Ltd, Tokyo, Japan) operating at an accelerating voltage of 100 kV. Ag-nps diameter was estimated by analyzing the TEM photos with ImageJ 1.42q software.

### Determination of liver function

Whole blood samples were collected from tail vein and allowed to clot followed by centrifugation at 1500 × g for 10 min, and then used for determination of liver function. Liver function was determined based on enzymatic analysis of aspartate aminotransferase (AST) and alanine aminotrasferase (ALT) activities [8,30]. AST and ALT activities were assayed by a biochemical blood analyzer (DRI-CHEM 3500 s, FUJIFILM, Japan).

### Histopathologic evaluation of liver injury and Ag-nps distribution

At each designated time points, rats were perfused under ether anesthesia with 0.9% normal saline and 4% paraformaldehyde through left ventricle. Liver tissues were removed and post-fixed in 4% paraformaldehyde for 24 h, followed by dehydratation with graded percentages of alcohol. After embedding in paraffin, the tissues were sectioned in 5-μm thickness and stained with hematoxylin and eosin (H&E) for histopathologic evaluation of liver injury. To assess distribution of Ag-nps in liver tissues, paraffin-embedded tissue sections were stained with silver enhancement method using HQ silver enhancement kit (Nanoprobes) [40]. Ag-nps distribution and liver injuries were examined under optical microscopy (ECLIPSE 80i, Nikon, Japan).

### Evaluation of autophagy

Autophagy was evaluated based on evidence of LC3-II aggregation and its protein expression in liver tissues [41]. LC3-II aggregation was determined by immunofluorescence staining. The deparaffinized tissues sections were incubated with hydrogen peroxide followed by incubation with serum blocking solution (10% normal goat serum, 1% bovine serum albumin and 1% Triton X-100 in 0.1 M PBS). Samples were then incubated with anti-LC3B antibody at 1:100 dilution for 2 h at room temperature followed by incubation overnight at 4 °C. After thorough rising the sections were incubated with FITC-conjugated goat anti-rabbit IgG antibody (1:500) for 1 h, following dehydration and mounting. Images were analyzed using a fluorescence microscopy (Zeiss AxioVert 200 M; Jena, Germany) equipped with a computer-controlled mechanical stage and a camera. Image acquisition was controlled by RSImage (Photometrics, Tucson, Ariz). For further quantification of autophagy, LC3-II protein levels were determined by Western blot analysis. Briefly, samples of liver homogenate containing 50 μg protein were denatured and subjected to sodium dodecyl sulfate (SDS)-polyacrylamide gel electrophoresis (PAGE). Proteins separated by SDS-PAGE and transferred onto polyvinylidene difluoride membranes by electro-blotting for 1 h (100 V). The membranes were then blocked with 5% nonfat milk in Tris-buffer saline (TBS). Membranes were washed with TBST (TBS containing 0.5% Tween 20), followed by incubation with primary antibody against LC3 (1:1000) at 4°C overnight. Subsequently, membranes were incubated with secondary antibody (1:10000) at room temperature for 1 h. β-actin was used as an internal housekeeping control. Protein bands were enhanced with chemiluminescence (Amersham), visualized on FUJI Medical X-ray film, and the relative densities were quantified. All values were normalized for β-actin expression.

### Analysis of apoptosis

Apoptosis was analyzed based on caspase-3 protein expression and terminal deoxynucleotidyl transferase dUTP nick end labeling (TUNEL) assay [42,43]. Caspase-3 protein expression was determined as described in the preceding paragraph for LC3 except that cleaved caspase-3 was used as a primary antibody and rabbit IgG, HRP-linked whole antibody as a secondary antibody. TUNEL assay was employed to detect apoptotic cells using In Situ Cell Death Detection Kit (Roche Applied Science). In brief, the deparaffinized sections (5 μm thick) were treated with 10 μg/ml proteinase K in 0.1 M PBS (pH 7.4) for 30 min at 37°C. Subsequently, these sections were incubated with 0.1 M citrate buffer (pH 6) under microwave irradiation. After thorough rinsing, samples were incubated with TUNEL reaction mixture for 1 h at 37°C in humidified chamber that contains TdT and TMR-dUTP. After washing, the fluorescence-labeled images were visualized by a fluorescence microscopy. Total nucleoli were identified based on DAPI staining. Percentages of TUNEL-positive cells, indicative of DNA damage, were calculated by dividing the number of TUNEL-positive cells by total number of nucleoli.

### Determination of ATP content

ATP content was quantified based on bioluminescence assay with recombinant firefly luciferase and its substrate _D_-luciferin [44]. The assays were performed using a commercially available ATP determination kit (Molecular Probes). Briefly, liver homogenates were added to a standard reaction solution containing firefly luciferase, _D_-luciferin and DTT. Under the effects of luciferase, the luminescence evoked by the interaction of ATP and luciferin was detected by a luminometer (HIDEX, Turku, Finland) and ATP content was calculated.

### Statistical analysis

All values were expressed as mean ± standard deviations. Data analysis and evaluation of statistical significance between the two groups of parameters were subjected to ANOVA followed by Tukey’s least significant difference procedure. Significant difference was accepted at P-values less than 0.05.

## Abbreviations

Ag-nps: Silver nanoparticles; ALT: Alanine aminotrasferase; AST: Aspartate aminotransferase; ATP: Adenosine triphosphate; DAPI: 2-(4-Amidinophenyl)-6- indolecarbamidine dihydrochloride; H&E: Ematoxylin&eosin; i.p.: Intraperitoneally; ICP-MS: Inductively coupled plasma-mass spectrometry; LC3: Microtubule- associated protein 1-light chain 3; PBS: Phosphate-buffered saline; ROS: Reactive oxygen species; SD: Sprague–Dawley; TEM: Transmission electron microscopy; TUNEL: Terminal deoxynucleotidyl transferase dUTP nick end-labeling.

## Competing interests

There are no financial or non-financial competing interests.

## Authors’ contributions

Conceived and designed the experiments: TYL, LJH, SIL and RCY. Performed the experiments: TYL and SIL. Analyzed the data: TYL and RCY. Contributed reagents/materials/analysis tools: TYL, MSL, LJH, SIL, LCL, ALK and RCY. Wrote the paper: TYL, MSL and RCY. All authors read and approved the final version of the manuscript.

## Supplementary Material

Additional file 1 **Transmission electron micrograph of hepatocyte in sham group.** TEM images of hepatocyte of liver tissues obtained from rats at day 1 following deionized water administration. Click here for file

Additional file 2 **Characterization of silver nanoparticles in Kupffer cells.** Transmission electron micrograph images on the deposition of silver nanoparticles in Kupffer cell of liver tissues obtained from rats at day 1 following Ag-nps administration. Tissues were processed for TEM evaluation as described under Materials and Methods. Accelerating voltages and magnifications were indicated at right lower corner. Scale Bar size represent 5 μm in panel A, 2 μm in panel B, 0.5 μm in panel C, and 0.2 μm in panel D. Panel B represents the enlargement of yellow square area indicated in panel A. Panel C represents the enlargement of yellow square area indicated in panel B. Panel D represents the enlargement of yellow square area indicated in panel C. White arrows in panel D indicate silver nanoparticle agglomerates. Click here for file

Additional file 3 **Transmission electron micrograph image of autophagic vacuoles in macrophage.** Ag-nps induced the formation of autophagic vacuoles in macrophage of rat liver tissues at day 1 following Ag-nps administration. Scale bar size represent 2 μm. Accelerating voltages and magnifications were indicated at right lower corner. Black arrows indicate autophagic vacuoles (AV). Click here for file

Additional file 4 **Serum bilirubin level following intraperitoneal exposure to Ag-nps in rats.** There were no significant differences between the treatment and the sham groups. Values were expressed as means ± SD (n = 8). Click here for file
