# Supplementary material for: Bioenergetic failure correlates with autophagy and apoptosis in rat liver following silver nanoparticle intraperitoneal administration
Source: Part Fibre Toxicol. 2013 Aug 19;10:40. doi: 10.1186/1743-8977-10-40 (PMC3765627; doi:10.1186/1743-8977-10-40)
Supplement: Additional file 1 — Transmission electron micrograph of hepatocyte in sham group. TEM images of hepatocyte of liver tissues obtained from rats at day 1 following deionized water administration. [file 1743-8977-10-40-S1.doc]

**Additional file 1**


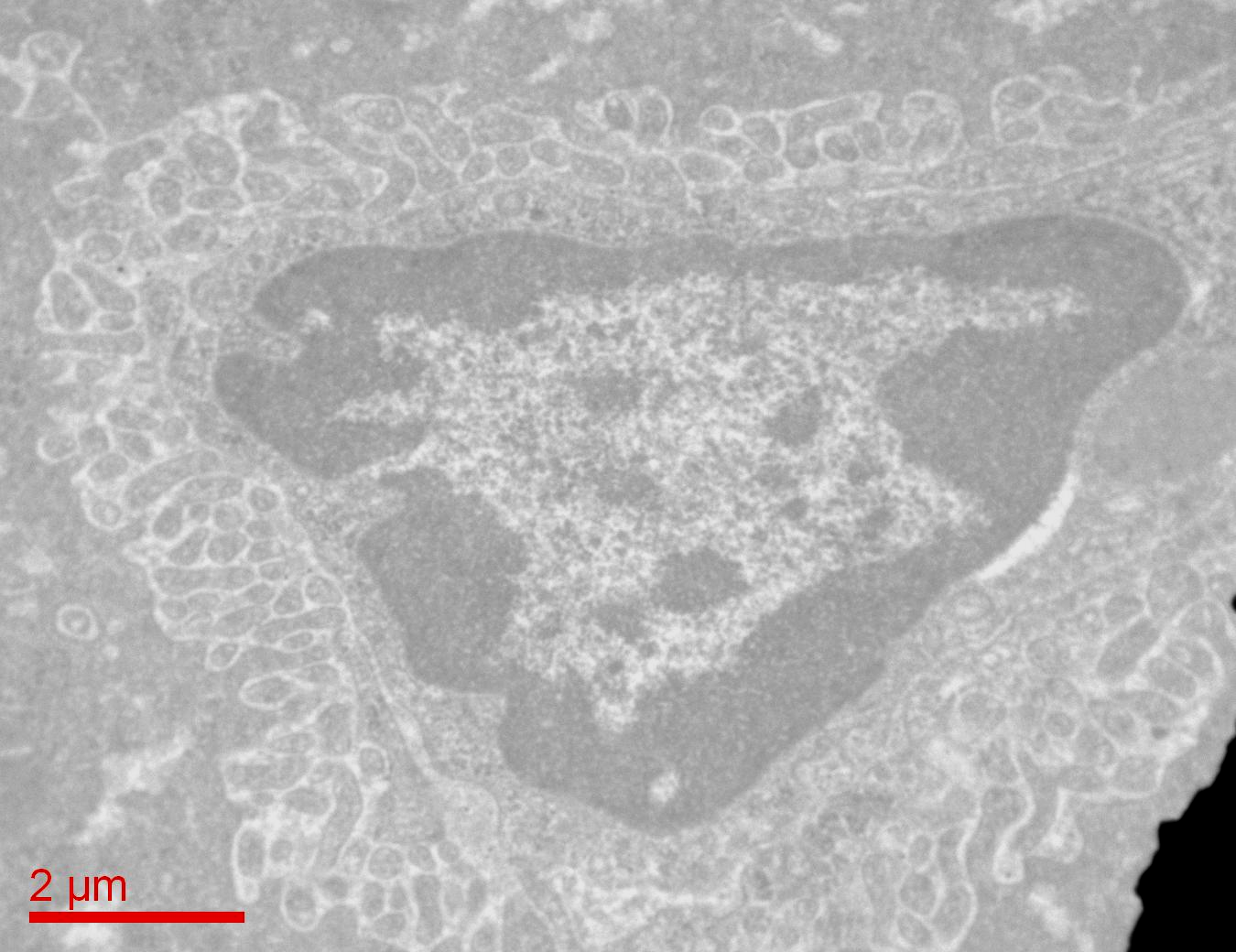


**Additional file 1 (PDF) - Transmission electron micrograph of hepatocyte in sham group.** TEM images of hepatocyte of liver tissues obtained from rats at day 1 following deionized water administration.
