# Supplementary material for: Bioenergetic failure correlates with autophagy and apoptosis in rat liver following silver nanoparticle intraperitoneal administration
Source: Part Fibre Toxicol. 2013 Aug 19;10:40. doi: 10.1186/1743-8977-10-40 (PMC3765627; doi:10.1186/1743-8977-10-40)
Supplement: Additional file 2 — Characterization of silver nanoparticles in Kupffer cells. Transmission electron micrograph images on the deposition of silver nanoparticles in Kupffer cell of liver tissues obtained from rats at day 1 following Ag-nps administration. Tissues were processed for TEM evaluation as described under Materials and Methods. Accelerating voltages and magnifications were indicated at right lower corner. Scale Bar size represent 5 μm in panel A, 2 μm in panel B, 0.5 μm in panel C, and 0.2 μm in panel D. Panel B represents the enlargement of yellow square area indicated in panel A. Panel C represents the enlargement of yellow square area indicated in panel B. Panel D represents the enlargement of yellow square area indicated in panel C. White arrows in panel D indicate silver nanoparticle agglomerates. [file 1743-8977-10-40-S2.doc]

**Additional file 2**


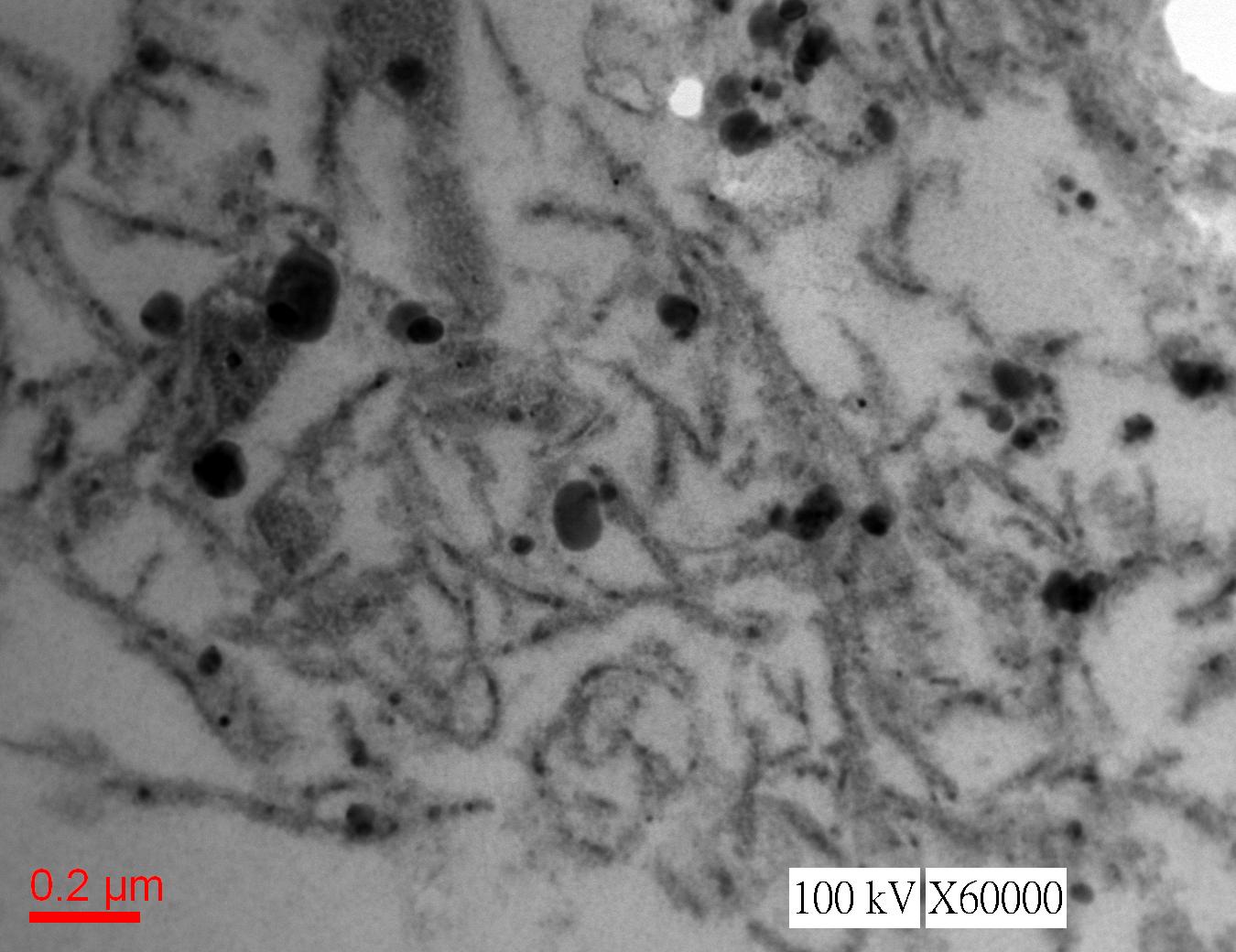

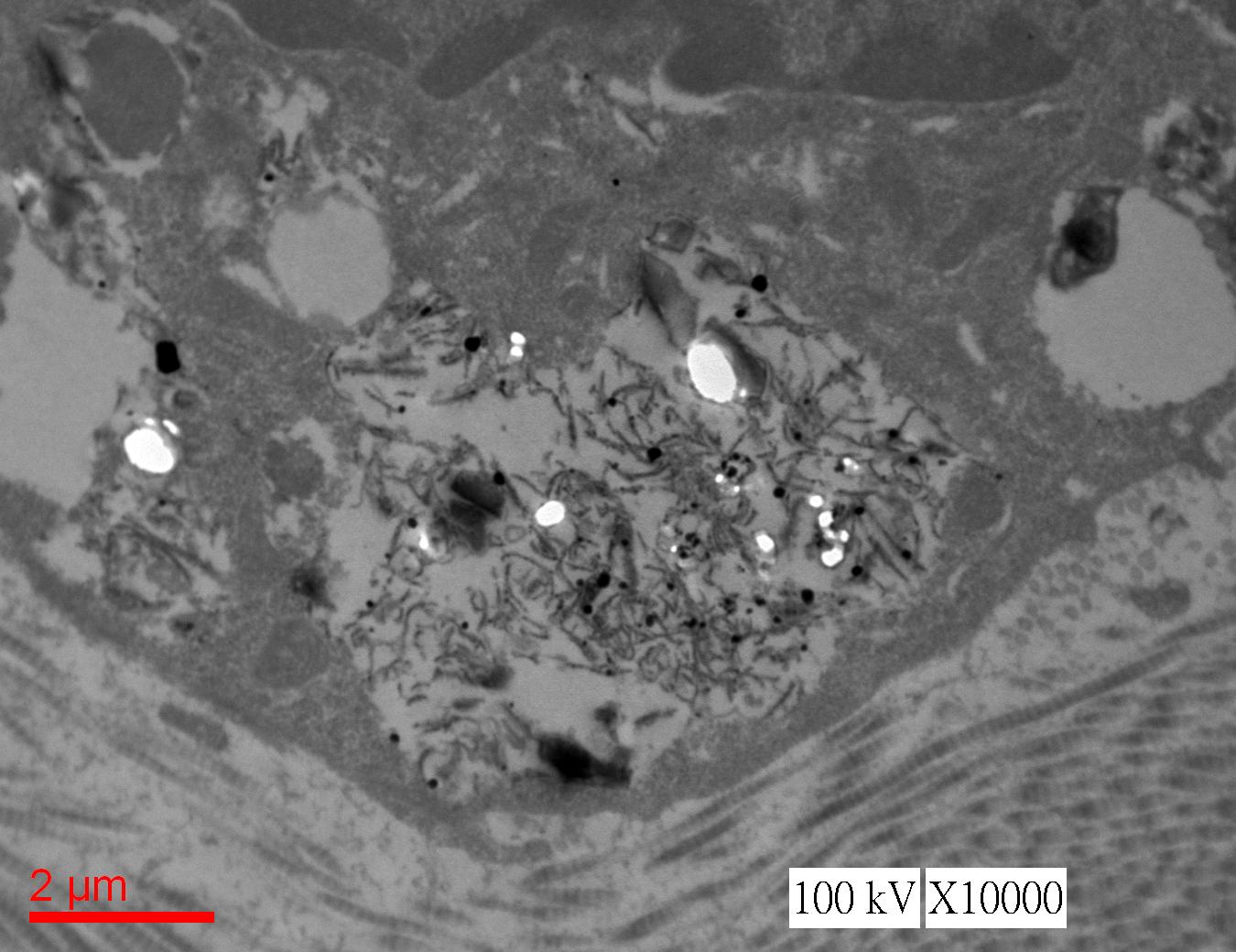

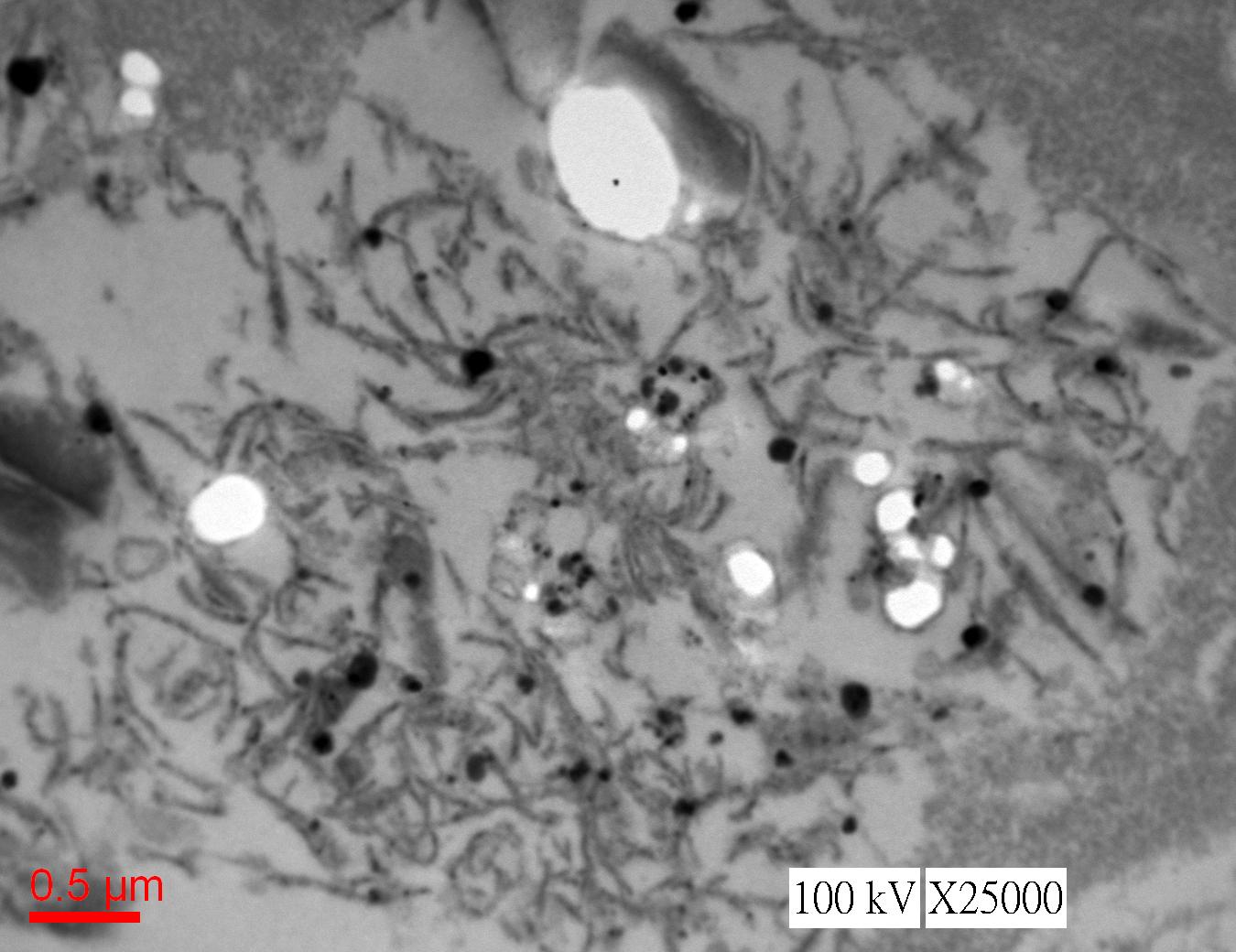

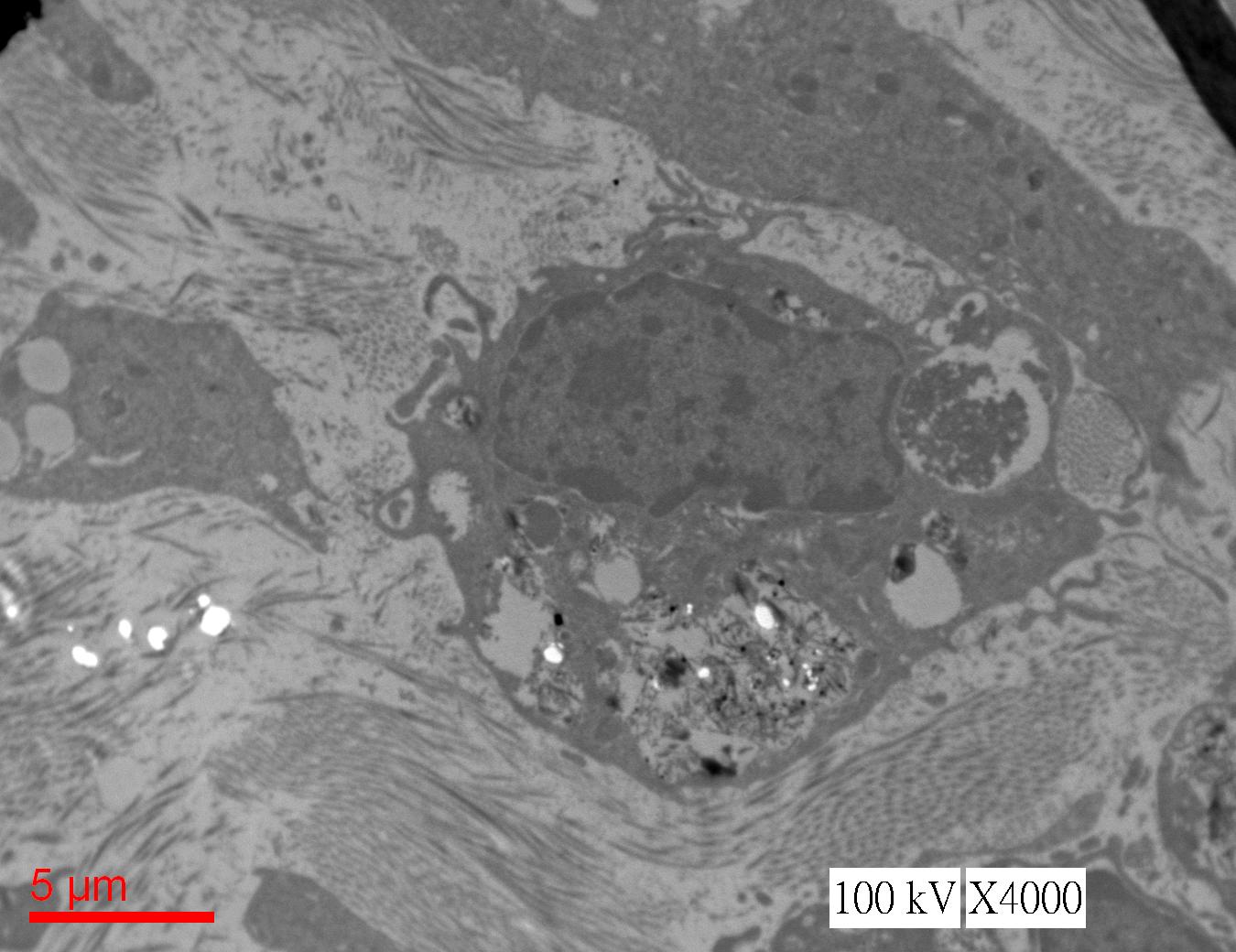


**A**

**B**

**C**

**D**

148nm

101nm

103nm

**Additional file 2 (PDF) - Characterization of silver nanoparticles in Kupffer cells.** Transmission electron micrograph images on the deposition of silver nanoparticles in Kupffer cell of liver tissues obtained from rats at day 1 following Ag-nps administration. Tissues were processed for TEM evaluation as described under Materials and Methods. Accelerating voltages and magnifications were indicated at right lower corner. Scale Bar size represent 5 μm in Panel A, 2 μm in Panel B, 0.5 μm in Panel C, and 0.2 μm in Panel D. Panel B represents the enlargement of yellow square area indicated in Panel A. Panel C represents the enlargement of yellow square area indicated in Panel B. Panel D represents the enlargement of yellow square area indicated in Panel C. White arrows in Panel D indicate silver nanoparticle agglomerates.
