# Supplementary material for: Bioenergetic failure correlates with autophagy and apoptosis in rat liver following silver nanoparticle intraperitoneal administration
Source: Part Fibre Toxicol. 2013 Aug 19;10:40. doi: 10.1186/1743-8977-10-40 (PMC3765627; doi:10.1186/1743-8977-10-40)
Supplement: Additional file 3 — Transmission electron micrograph image of autophagic vacuoles in macrophage. Ag-nps induced the formation of autophagic vacuoles in macrophage of rat liver tissues at day 1 following Ag-nps administration. Scale bar size represent 2 μm. Accelerating voltages and magnifications were indicated at right lower corner. Black arrows indicate autophagic vacuoles (AV). [file 1743-8977-10-40-S3.doc]

**Additional file 3**


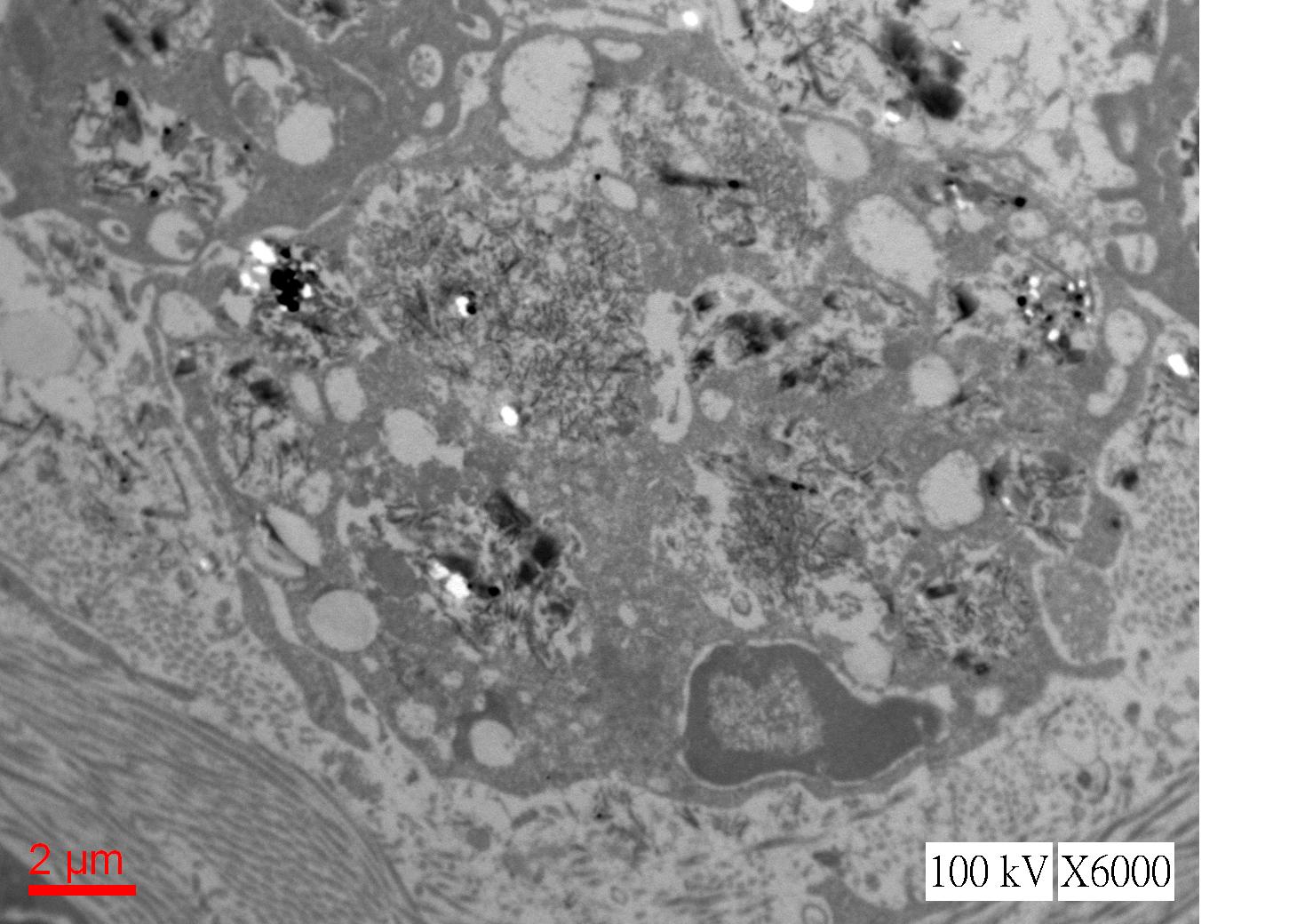


**AV**

**AV**

**Additional file 3 (PDF) - Transmission electron micrograph image of autophagic vacuoles in macrophage.** Ag-nps induced the formation of autophagic vacuoles in macrophage of rat liver tissues at day 1 following Ag-nps administration. Scale bar size represent 2 μm. Accelerating voltages and magnifications were indicated at right lower corner. Black arrows indicate autophagic vacuoles (AV).
