# Supplementary material for: Bioenergetic failure correlates with autophagy and apoptosis in rat liver following silver nanoparticle intraperitoneal administration
Source: Part Fibre Toxicol. 2013 Aug 19;10:40. doi: 10.1186/1743-8977-10-40 (PMC3765627; doi:10.1186/1743-8977-10-40)
Supplement: Additional file 4 — Serum bilirubin level following intraperitoneal exposure to Ag-nps in rats. There were no significant differences between the treatment and the sham groups. Values were expressed as means ± SD (n = 8). [file 1743-8977-10-40-S4.doc]

**Additional file 4**

**Time after treatment**

Day1

Day4

Day7

Day10

Day30

Sham

0

0.05

0.1

0.15

0.2

0.25

0.3

0.35

0.4

**Total Bilirubin (mg/dl)**

Sham group

Ag-nps group

**Additional file 4 (PDF) - Serum bilirubin level following** **intraperitoneal exposure to Ag-nps in rats.** There were no significant differences between the treatment and the sham groups. Values were expressed as means ± SD (n=8).
